# A Large-Scale Distribution of Milk-Based Fortified Spreads: Evidence for a New Approach in Regions with High Burden of Acute Malnutrition

**DOI:** 10.1371/journal.pone.0005455

**Published:** 2009-05-06

**Authors:** Isabelle Defourny, Andrea Minetti, Géza Harczi, Stéphane Doyon, Susan Shepherd, Milton Tectonidis, Jean-Hervé Bradol, Michael Golden

**Affiliations:** 1 Médecins sans Frontières, Paris, France; 2 University of Aberdeen, Aberdeen, Scotland; AgroParisTech, France

## Abstract

**Background:**

There are 146 million underweight children in the developing world, which contribute to up to half of the world's child deaths. In high burden regions for malnutrition, the treatment of individual children is limited by available resources. Here, we evaluate a large-scale distribution of a nutritional supplement on the prevention of wasting.

**Methods and Findings:**

A new ready-to-use food (RUF) was developed as a diet supplement for children under three. The intervention consisted of six monthly distributions of RUF during the 2007 hunger gap in a district of Maradi region, Niger, for approximately 60,000 children (length: 60–85 cm). At each distribution, all children over 65 cm had their Mid-Upper Arm Circumference (MUAC) recorded. Admission trends for severe wasting (WFH<70% NCHS) in Maradi, 2002–2005 show an increase every year during the hunger gap. In contrast, in 2007, throughout the period of the distribution, the incidence of severe acute malnutrition (MUAC<110 mm) remained at extremely low levels. Comparison of year-over-year admissions to the therapeutic feeding program shows that the 2007 blanket distribution had essentially the same flattening effect on the seasonal rise in admissions as the 2006 individualized treatment of almost 60,000 children moderately wasted.

**Conclusions:**

These results demonstrate the potential for distribution of fortified spreads to reduce the incidence of severe wasting in large population of children 6–36 months of age. Although further information is needed on the cost-effectiveness of such distributions, these results highlight the importance of re-evaluating current nutritional strategies and international recommendations for high burden areas of childhood malnutrition.

## Introduction

There are 146 million underweight (stunted and/or wasted) young children in the developing world. Wasting rates in highly affected countries often exceed 10% and 40% of all under five children are stunted [1]. In children, stunting and wasting are indicators of type II nutrient deficiencies in the diet, which have systemic effects on health and survival beyond growth failure and emaciation [2]. There is a strong association between malnutrition and mortality, which increases with declining anthropometrical status [3]. Severe acute malnutrition (SAM), marasmus and kwashiorkor, is the most severe and life-threatening presentation of childhood malnutrition. Childhood malnutrition is generally defined by anthropometric indicators: weight-for-height index of <−3 z-scores or mid-upper arm circumference (MUAC) of <110 mm. However, deficiencies undetected by anthropometric measurements also impact on morbidity and mortality [4]. Overall, malnutrition is implicated in between 2.2 and 5 millions deaths in children under five every year [5], [6]. Despite global efforts to meet the Millennium Development Goal of halving the prevalence of underweight children under 5 by 2015, underweight rates are rising in many Sub-Saharan African countries and remain high in large parts of South Asia [7].

**Figure 1:**
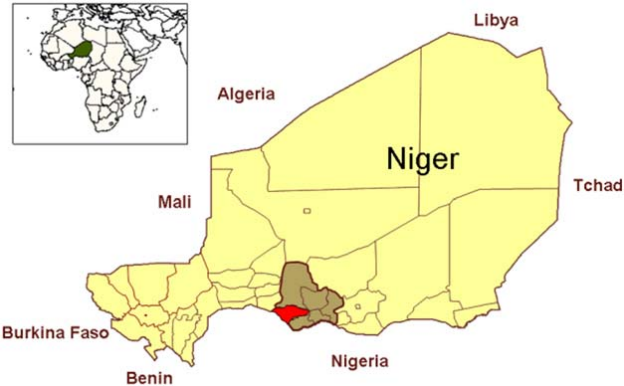
Map of Niger with Maradi region (brown) and Guidam Roumdji district (red).

In Niger, over the past decade, stunting has increased from 27% in 1992 to 41% in 1998 among children under three [8], [9]. In 2006, the Niger national Demographic and Health Survey estimates wasting and stunting prevalence among children under five at 10.3% and 50%, respectively; Niger has one of the highest under five child mortality rates in the world, at 198 per 1000 live births. Since 2001, Médecins sans Frontières (MSF), in collaboration with the Ministry of Health, has run a therapeutic feeding program in Maradi region (Figure 1). Up to 60% of children under five in Maradi are stunted, 43.6% are severely stunted, and under 5 child mortality is above the national average, at 231 deaths per 1000 [10]. Wasting and mortality rates reach particularly high levels in children less than two years of age [10]. Each year, tens of thousands of children with SAM are admitted to therapeutic feeding centres in the Maradi area.

Elsewhere, outpatient management of childhood malnutrition using therapeutic fortified spreads (ready-to-use therapeutic food or RUTF, Plumpy'nut®, Nutriset, Malaunay, France) has permitted both continuous increases in program capacity and coverage [11], and the extension of treatment to children with less severe stages of acute malnutrition [12].

In Niger, program admissions increase in the “hunger gap”, the long lean season from May to October before the harvest. This was particularly dramatic in 2005, when millet prices reached levels inaccessible to large segments of the population. During this time, MSF admitted over 40,000 severely malnourished children in Maradi region alone [13], [14]. In 2006, MSF extended the use of RUTF to 60, 000 moderately malnourished children. There were extremely high cure and low default rates, compared to results typically obtained with the standard treatment of moderate malnutrition with fortified blended flours [15]. The use of RUTF for moderately malnourished children flattened the typical seasonal rise of admissions for severe acute malnutrition [16].

However, the treatment of individual cases of malnutrition is expensive and difficult to provide on a large-scale, particularly in countries where resources, both financial and human, are limited. Moreover, beyond treating or preventing SAM, it is clear that in Maradi region underlying nutritional deficiencies affect essentially the whole population of young children. In fact, there is a shift in the entire population's growth curve away from normal, as reported in a survey conducted in Niger in 2005 [17]. When growth failure affects such large segments of a given population, it seems pertinent to devise intervention strategies at population level to address the problem [18], [19].

In large nutritional emergencies and in regions with chronic high burdens of high childhood malnutrition such as Maradi, we wanted to examine whether it would be more feasible and effective to aim for blanket coverage with a nutritional supplement, which can prevent the development of stunting and wasting. In a region with such a high burden of acute malnutrition, it is unrealistic to expect health services to handle the enormous underlying burden of untreated malnutrition and associated morbidity.

Previous studies have already shown the efficacy of the use of fortified spreads as a supplement to traditional complementary food on growth among non-malnourished and moderately malnourished infants [20], [21]. Among mildly wasted children in Malawi, Patel et al. demonstrated greater weight gain and recovery in children receiving RUTF than in those receiving fortified flour [22]. Phuka et al. reported that severe stunting was significantly lower among infants receiving a supplementation with a ready-to-use fortified spread than those receiving fortified flour [23]. Furthermore, this effect on height was sustained over a 24-months observation period and the children in the fortified spread cohort continued to display improved weight gain after the supplementation phase ended [24]. Moreover, previous evidence suggests that preventive models of delivering nutritional interventions are more effective for the reduction of childhood undernutrition than traditional recuperative programs [25].

In 2007, MSF implemented a new strategy in its Maradi program with the intention of preventing the seasonal rise in the incidence of severe acute malnutrition while improving the nutritional status of an entire population of high-risk young children. Central to the strategy was a blanket distribution of a fortified spread to approximately 60,000 children aged 6 to 36 months in the district of Guidam Roumdji, Maradi region. At each monthly distribution, children were also screened for SAM and referred for therapeutic treatment if indicated. In an effort to detect children at an earlier stage of acute malnutrition, in 2007 also MSF adopted more sensitive admission criteria for severe acute malnutrition (WHO_2006_ growth standards) [26]–[28].

## Methods

In late 2006, a new ready-to-use food (RUF) was developed as a dietary supplement for children under the age of three (Plumpy'doz®, Nutriset, Malaunay, France). Plumpy'doz® is an energy-dense, nutrient-enriched paste made of milk powder, peanuts, oil and sugar, whose composition is based on therapeutic products, such as F-100 and Plumpy'nut®, but with higher level of vitamin and mineral fortification. In contrast to therapeutic diets that provide all of the child's calorie and nutrient needs, this RUF is designed to be consumed in small amounts (47 g or 3 tablespoons/day) as a supplement to the infant or toddler's daily diet of breast-milk and family foods (Table 1).

**Table 1:** Recommended Daily Intakes for infants and nutritional composition of RUF (Plumpy'Doz®)

	Recommended Daily Intakes * for a healthy 12–36 months child	RUF (3 spoons/day)
**KCAL**	**1022**	**250**
**Grammes**		**47**
**Proteins** g	**13**	**5.9**
**Fat** g	**35**	**16**
**Calcium** mg	**500**	**387**
**Phosphorus** mg	**460**	**275**
**Zinc** mg	**2.4–8.3**	**4**
**Copper** mg	**0.34**	**0.3**
**Iron** mg	**3.9–11.6**	**9**
**Iode** µg	**90**	**55**
**Selenium** µg	**17**	**17**
**Vit A** µg	**400**	**400**
**Vit B1** (Thiamine) mg	**0.5**	**0.5**
**Vit B2** (Riboflavine) mg	**0.5**	**0.5**
**Vit B6** mg	**0.5**	**0.5**
**Vit B12** µg	**0.9**	**0.9**
**Vit C** mg	**30**	**30**
**Vit B9** (Folic Acid) µg	**150**	**160**
**Vit B3** (Niacin) mg	**6**	**6**

* Vitamin and Mineral requirements in human nutrition, WHO & FAO, 2^nd^ edition 2004

The intervention in 2007 consisted of a monthly distribution of 4 pots of RUF (325 g/pot) to each child from May to October 2007. Participation was voluntary. Caregivers were instructed to give three tablespoons of RUF to each beneficiary child each day so that one pot would last for one week. All children between 6 and 36 months of age and resident in the district of Guidam Roumdji between the 14^th^ and the 16^th^ of April 2007 (registration days) were considered eligible. As precise age is usually unknown in this context, height was taken as a proxy of age and children were registered for distribution with a height between 60 and 85 cm. We selected 60 cm due to the high prevalence of stunting, as the proxy length for a child 6 months old. This ensured that children with a lower height for age were not missed in the preventive distribution. After initial registration, children were not recruited to the cohort if they reached 60 cm of length (6 months) or dropped from the cohort if they exceeded 85 cm of length (36 months).

The district of Guidam Roumdji (4500 km^2^) was divided into 7 distribution zones. 52 distribution sites were chosen in the district (between 6 and 9 per zone), generally close to a health centre or health post. The centres were selected geographically so that the majority of families would be within 7 km of a distribution site. A distribution team of 5 persons covered each zone. Initially, teams visited each village and explained the program with the mediation of village leaders. The RUF was presented as a supplement in addition to the foods young children typically eat and not a replacement for breastfeeding. Families were told that the RUF is not appropriate for infants less than 6 months of age.

During registration, each mother was given a registration booklet with tear-out tickets, one for each month's distribution. These were collected at each distribution and used to count the number of beneficiaries. The average number of mothers and children attending a given centre on the day of distribution was 1226 (358 to 3074). Teams conducted the distribution over 9 consecutive days each month, each team covering one site per day. The rest of the month, the distribution staff was engaged in community mobilization.

At each distribution, all children over 65 cm had their Mid-Upper Arm Circumference (MUAC) recorded. Children whose height was suspected of being less than 65 cm were measured with a length board before the MUAC was taken. Any child whose MUAC was less than 110 mm was referred for therapeutic feeding.

All children admitted to the MSF therapeutic feeding program were medically assessed on a weekly basis and treated with RUTF (1000 kcal/day for children <8 kg and 1500 kcal/day for children >8 kg). Children with severe co-morbidities, nutritional oedema extending beyond the feet or a negative appetite test, were admitted for inpatient care. The appetite test is an integral part of the clinical assessment of SAM at the outpatient centre; the caregiver is given a sachet of RUTF and instructed to feed the child under observation, in a calm setting. The child must consume 30–40 grams of RUTF over a 30-minute period in order to be a candidate for out-patient care. Children unable to consume this amount are consider to be anorexic and are referred to inpatient care.

Upon admission to the therapeutic program, the caregiver was asked whether the child was receiving RUF via the preventive distribution. Children already under treatment in the therapeutic feeding program continued to receive the RUF ration during distribution. Mothers of those children were instructed not to give the RUTF and the RUF together; they were advised to save the RUF ration until their child was discharged from the therapeutic program. MUAC was the only anthropometric measurement taken during the monthly RUF distributions. We used aggregated admissions data to the therapeutic feeding programs in Maradi from 2002–2005 to obtain a model of expected seasonality. The year 2006 was omitted because early treatment of large numbers of moderately wasted cases had largely decreased the seasonal rise in severe cases. The absolute numbers of beneficiaries attending the 2007 RUF distribution were counted from the tally sheets of the distribution teams.

As this analysis reports on program monitoring data, ethical committee approval was not sought nor was individual written consent obtained.

## Results

A total of 65,085 children were registered initially for inclusion in the distribution program. 62,922 beneficiaries attended the first RUF distribution (according to the number of tickets collected). Remarkably few of them failed to attend subsequent distributions with nearly the same number of beneficiaries attending all six distributions (decrease of 1.6% from first to last distribution).

**Figure 2:**
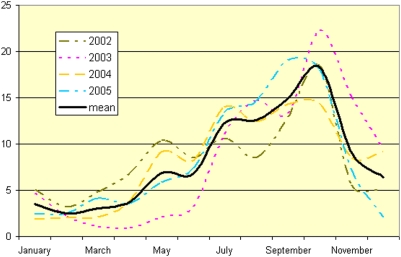
Monthly percent of annual admissions from Guidam Roumdji (<70% NCHS) to MSF therapeutic feeding programs in Maradi, Niger, 2002–2005.

Data presented in Figure 2 shows the number of children admitted for severe acute malnutrition (W/H<70% NCHS) for a given month as a proportion of the yearly total for the years 2002–2005. Each year, the fewest children were admitted in February; monthly admissions then rose steadily to reach a peak in October, followed by a steady decline to February of the next year.

**Figure 3:**
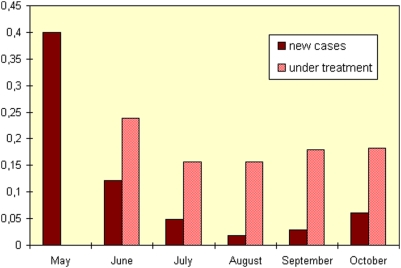
Prevalence of severe wasting (by MUAC<110 mm) in Guidam Roumdji, Maradi, Niger, 2007.

The results of MUAC screening performed every month during the 2007 distribution show that, despite the annual hunger gap season, the prevalence of children with MUAC<110 mm between May and August decreased by half, rising slightly in September and October (data not shown). This prevalence includes children that had been identified in previous months and were already under active treatment for their malnutrition, at the time of the screening. In Figure 3 those children with MUAC<110 mm that are new cases are presented separately from those already under treatment. The incidence of SAM fell and remained at extremely low levels. The seasonal rise in admissions for SAM (W/H<70% NCHS) described in Figure 2 is not confirmed by the incidence of SAM (MUAC<110 mm) observed in 2007 (Figure 3). The expected rise in new cases during the hunger gap period in 2007 was not only arrested, but reversed during the period of blanket distribution of RUF.

**Figure 4:**
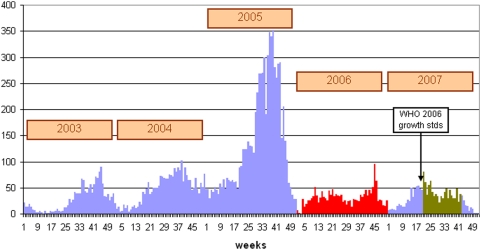
Total number of severely malnourished children (<70% NCHS or MUAC or oedema) from Guidam Roumdji admitted to the MSF treatment program in Maradi, Niger 2003–2007 (in red: treatment of moderate malnutrition with RUTF; in dark green: 6-months RUF distribution).

Figure 4 shows the trend in admissions for SAM in Guidam Roumdji district for the years 2003–2007. It should be noted that although not measured by survey, coverage of the program almost definitely increased from year to year as the number of structures and human resources increased over time (for example, 3 outpatient centres for therapeutic feeding in 2003 compared to 9 outpatient centres in 2007).

Table 2 provides data for children from Guidam Roumdji admitted to the therapeutic program, by their participation in the monthly RUF distribution. The proportion of complicated cases of malnutrition requiring intensive care was lower among children registered for RUF distribution (7.8%, p = 0.005) than among those not registered for distribution (9.9%). The proportion of malnourished children treated exclusively in outpatient facilities was higher among beneficiaries of the preventive distribution (84.2%, p<0.0001) than among children from Guidam Roumdji not registered for distribution (80.5%).

**Table 2:** Type of care and treatment response of children 6–36 months from Guidam Roumdji, by beneficiaries of RUF distribution, Maradi, Niger 2007

	RUF	No RUF	P value
**N**	3,362	2,949	
**Type of Care:**			
Outpatient only	84.2 (2832)	80.5 (2373)	<0.0001
Inpatient only	1.7 (58)	3.7 (108)	<0.0001
Requiring ICU	7.8 (264)	9.9 (291)	0.005
**Outcome:**			
Cured	92.3 (3054)	90.1 (2621)	0.003
Died	1.8 (60)	2.2 (65)	0.237
Default	4.7 (156)	6.0 (174)	0.026
Non-respondent	1.1 (37)	1.4 (41)	0.302
**Treatment Response:**			
Weight Gain (g/kg /day)	5.1±4.6	5.5±4.7	0.005
Length of Stay (days)	44.4±29.8	44.4±29.6	0.951

Among children receiving a preventive distribution of RUF, cure rate was higher (92.3%, p = 0.003) and default rate was lower (4.7%, p = 0.026), when compared with rates of children not receiving any preventive distribution (cure rate: 90.1%; and default rate: 6%).

## Discussion

A distribution of milk-based fortified spreads targeted to children 60–85 cm of length (6–36 months of age) has been shown here to be associated with a reduction in the prevalence and incidence of SAM as measured by the MUAC in a large population. In addition, beneficiaries of the RUF distribution were more often admitted to the therapeutic feeding program with non-complicated forms of malnutrition; thus, they were less likely to require hospitalization, and they exhibited better outcomes. Reducing the annual number of children with SAM is crucial in high burden areas of malnutrition.

It is particularly interesting to focus on the years 2006 and 2007. The blanket distribution strategy in 2007 with a small daily supplement of RUF had essentially the same effect on flattening out the usual seasonal rise in admissions for SAM as the individualized treatment of almost 60,000 children with moderate wasting within an intensive therapeutic program. These results highlight the importance of re-evaluating current nutritional strategies and international recommendations for high burden areas of childhood malnutrition.

There are limitations to these encouraging results, which require comment. First, attendance to distribution could be slightly overestimated as it has been calculated based on the number of tickets collected. In fact, mothers were required to attend each distribution with their child, but it was not possible to verify identity and the RUF was given irrespective of the child present, as long as he appeared to be within the target age range. It is also important to note that the decline in the number of children with MUAC<110 mm can be partially explained by the natural expected increase in MUAC with age. Also, we did not measure dietary intakes at baseline or during the intervention. We therefore did not have information on average energy intake or the macro- and micronutrient composition of baseline diets, or change in diets during the intervention, to indicate whether RUF supplemented or displaced usual intake. However, previous findings suggest that complementary feeding of infants with fortified spread likely do not replace other foods and breast milk intake [29], [30]. We also did not monitor the sharing or selling of the RUF within the household and there is a clear need for studies looking at how these products are actually used.

The suggested protective effect of the intervention is consistent with RUF use in a variety of settings [23], [31]–[35]. These results add to the growing evidence for the potential of direct nutritional intervention to mitigate the burden childhood malnutrition in regions with high rates of malnutrition and mortality. They are further evidence of the importance of dietary nutritional deficiencies in the development of malnutrition in young children, particularly in large, food insecure, rural poor populations subsisting on a diet of limited diversity, low consumption of animal source foods, and poor access to affordable, high quality nutrient-dense foods for their children. During the blanket distribution, there were no additional interventions that could have had an impact on SAM incidence. In particular, education, vaccination, vitamin A capsule distribution, water and sanitation programs, breast-feeding promotion, health clinic activities or curative services did not change. Although food availability can fluctuate from year-to-year, cereal prices in 2007 were very similar to those in 2006 [36]. The only change was the blanket distribution of this RUF. These results should provoke a re-evaluation of the relative roles of food, education, care and infection in the aetiology of malnutrition. They should also replace the notion that “food” is simply a question of supplying sufficient energy and protein. Providing all essential nutrients should be ensured in the choice of preventive and curative strategies.

Perhaps the most remarkable finding arising from this study is the consistently high number of beneficiaries during the six-month distribution. The mothers and children showed that, repeatedly, they considered a walk of up to 14 km to get the product a worthwhile investment of their time and energy. They reported that the children's skin became more supple and “bright”, that their appetites improved and that they were more active. Whatever the objective data on severe malnutrition, the clear endorsement by the community is a measure of the value that the recipients placed upon the program and their judgment, as the primary caregivers to their children, of the effect of the program.

Although these results are encouraging, it is important to confirm these findings in other settings and populations, ideally through comparative and randomized trials. Further large-scale trials are needed to document whether there are other critical benefits of fortified spreads on health, growth, mental development, immunocompetence and other physiological parameters that directly affect the survival and quality of life of young children in such high-risk situations.

The results obtained in the MSF Niger programs in 2006 and 2007 challenge us to find ways of ensuring that children in impoverished societies have access to food of high nutritional value in sufficient amounts for them to grow and develop normally. The large-scale distribution of RUF is one effective way to improve food aid and nutritional programming for high-risk populations. On feasibility and costs issues, programs like those implemented in Guidam Roumdji deserve a few remarks. Although both the 2006 and 2007 programs are arguably similar in terms of their benefits in reducing the prevalence of severe malnutrition, there are differences in their costs. The preventive distribution described here was 29% higher in terms of total costs (2.7 million euros) than the 2006 program (2.1 million euros), but the percentage of total costs given directly to the beneficiaries was much higher. In 2006, 35% of total expenses were allocated to RUTF given to families and 38% to staff related costs; in 2007, 77% of total expenses covered RUF and 15% the staff (MSF internal report). Moreover, in 2007, twice as many children received RUF. If the cost of the RUF were to decrease, the 2007 program would become potentially less expensive than even the 2006 program. Further, both programs are far beyond usual budgets dedicated to the treatment of underweight young children. Besides reducing the price of RUF, only a drastic increase of available funds for massive and early treatments of malnutrition cases could give a future to such programs. Further information is needed on the feasibility and cost-effectiveness of such a distribution, as RUF products are still more expensive than existing fortified flours. Further research is needed to determine precisely the minimum dose of RUF required as reductions in dose may reduce costs significantly, thereby leading to a more affordable and sustainable program.

In the context of the current global food crisis, policy makers have to consider the limits of existing food aid practice that does not provide food nutritious enough to meet the specific needs of young, growing children. The results presented here along with the growing body of evidence on direct food interventions for young children should prompt those who set food aid policy and practice to urgently implement innovative and large-scale programs specifically designed for those who, when resources are stretched thin, always pay the highest price: young children less than 3 years of age.
